# Intra-aortic balloon pump still has a role in late-onset myocardial infarction complicated by ventricular septal rupture with intractable heart failure: a case report

**DOI:** 10.1186/s13256-023-04284-3

**Published:** 2024-01-07

**Authors:** Mochamad Yusuf Alsagaff, Oky Revianto, Yan Efrata Sembiring, Muhammad Insani Ilman, Ryan Enast Intan

**Affiliations:** 1 Department of Cardiology and Vascular Medicine, Faculty of Medicine, Universitas Airlangga-Dr. Soetomo General Academic Hospital, Surabaya, East Java 60286 Indonesia; 2 Department of Cardiovascular Thoracic Surgery, Faculty of Medicine, Airlangga University-RSUD Dr. Soetomo General Hospital Surabaya, Surabaya, East Java 60286 Indonesia

**Keywords:** Intra-aortic balloon pump, Post myocardial infarction ventricular septal rupture, Intractable heart failure, Delayed surgical repair, Preoperative optimization

## Abstract

**Background:**

The current guidelines have discouraged the routine use of intra-aortic balloon pump (IABP) in cardiogenic shock complicating acute coronary syndrome (ACS). Since then, the trend of IABP utilization in ACS has been declining. Nevertheless, the guidelines still preserve the recommendation of IABP use in hemodynamic instability or cardiogenic shock caused by post myocardial infarction (MI) ventricular septal rupture (VSR).

**Case presentation:**

A 46-years-old diabetic Southeast Asian female was referred from a peripheral facility with intractable heart failure despite treatment with vasoactive agents and diuretics for five days. The ECG suggested a recent anteroseptal myocardial infarction with normal high-sensitivity troponin-I value. The echocardiography detected a regional wall motion abnormality and a 10 mm wide ventricular septal defect. Invasive coronary angiography revealed a severe two-vessel coronary artery disease. We planned a delayed surgical strategy with preoperative optimization using IABP as a bridge to surgery. IABP implantation followed by significant hemodynamic improvement and rapid resolution of heart failure without any inotrope support. Afterwards, coronary artery bypass grafting (CABG) and VSR surgical repair were performed. We safely removed IABP on the third postoperative day with proper weaning and minimal vasoactive support.

**Conclusion:**

We report a case where IABP still provided benefits for a patient with intractable heart failure caused by undetermined onset MI complicated by VSR. The use of IABP in such a case is in accordance with the recommendation of the current guidelines. Several studies showed that IABP use during preoperative optimization in the case of post-MI VSR was associated with survival benefits.

## Introduction

Intra-aortic balloon pump (IABP) has been the most employed mechanical circulatory support (MCS) device for the past half-century [1, 2]. It provides hemodynamic support for various clinical conditions, ranging from prophylactic measures for high-risk invasive coronary artery procedures to managing cardiogenic shock or advanced heart failure [2]. However, its utilization in recent years has been shown to be progressively reduced, particularly in acute myocardial infarction (AMI) [3].

IABP use in cardiogenic shock complicating AMI was formerly assigned to a Class I recommendation [4–6]. This recommendation mainly was based on observational studies. However, after the publication SHOCK-II trial, a multi-centre randomized clinical trial (RCT) with 600 subjects [7], routine use of IABP in cardiogenic shock complicating AMI had been downgraded to a Class III recommendation [8]. The trial showed that IABP did not significantly reduce 30-day mortality [7]. Nonetheless, IABP use in hemodynamic instability or cardiogenic shock due to mechanical complications of AMI is still preserved as a Class IIa recommendation (level of evidence C) [8].

Ventricular septal rupture (VSR) is a lethal mechanical complication of AMI [9–11]. The current guidelines suggested early surgical repair as an urgent treatment [8, 12]. However, delayed surgical strategy may be considered in patients who respond well to heart failure therapy, allowing infarcted tissue maturation for better surgical outcomes [8, 13]. Preoperative optimization of the already poor cardiac function and hemodynamic status during the deferred period is a serious challenge [14]. Various types of MCS have been shown to be feasible and effective during the preoperative period [15–17]. However, according to the current guidelines, the widely used IABP is still the first-line MCS in this clinical setting [8, 18]. It is also the simplest, safest, cheapest, and most studied MCS [2, 16].

We report a case that demonstrated IABP preoperative roles in the treatment of a patient with an intractable heart failure caused by VSR complicating a late-onset AMI who was planned to undergo coronary artery bypass grafting (CABG) and surgical repair of VSR. We will discuss whether IABP use in this clinical setting is still relevant.

## Case presentation

A 46-year-old Southeast Asian female was referred to our institution with persistent hypotension and intractable heart failure after five days of care in a peripheral hospital. In the peripheral hospital, the patient was initially treated for decompensated heart failure. Her condition worsened despite optimum available pharmacological treatment had been administered. The local physicians decided to refer the patient as soon as they suspected a concurrent ventricular septal defect.

The patient’s consciousness was still intact on admission. She complained of shortness of breath—especially when assuming a supinated position—excessive fatigue, and progressive legs swelling within the last month. There was no complaint of angina prior to or during hospitalization in the peripheral hospital. There was a history of poorly treated Type 2 Diabetes Mellitus for more than ten years. The presence of the other risk factors was denied.

The patient’s vital signs on arrival at the emergency department were as follows: blood pressure (BP) of 107/69 mmHg supported with continuous infusion of dobutamine 3 mcg/kg/min and norepinephrine 0.05 mcg/kg/min; heart rate (HR) was ranged between 95 to 115 beats/min; respiratory rate (RR) of 24 breaths/min; afebrile (36.9 °C); and peripheral oxygen saturation level (SpO2) of 96% aided by 4 L/min nasal cannula oxygen supplementation. Head and neck assessment showed anaemic conjunctiva and distended jugular vein. Chest auscultation revealed rales at the base of both lungs and a holosystolic murmur at the apex of the heart. There were ascites and lower limb oedema.

**Fig. 1:**
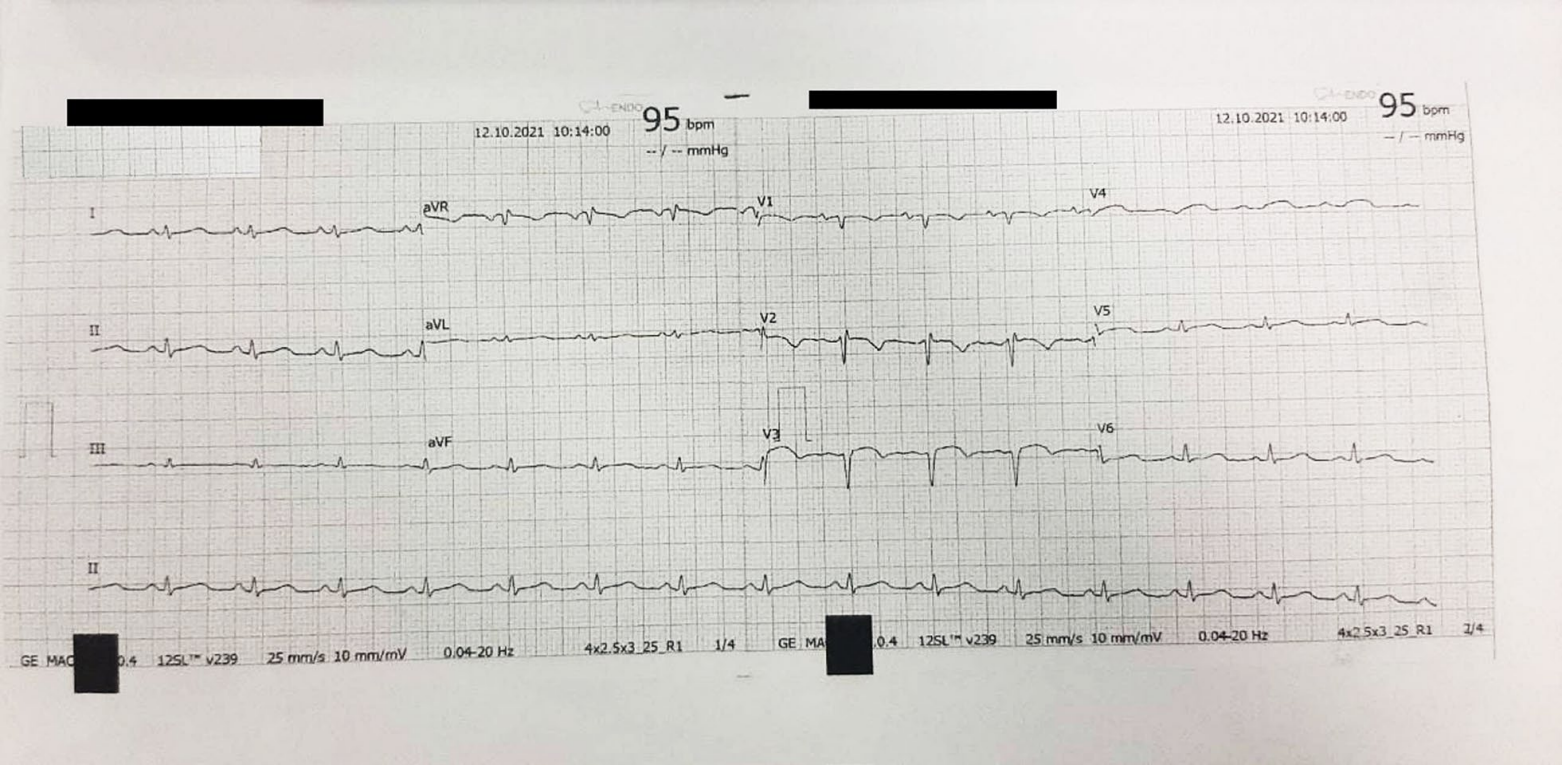
Electrocardiogram showed a QRS low voltage that may suggest loss of viable myocardium. Lead V1-V4 showed QS pattern suggesting a previous myocardial infarction at anteroseptal region

**Fig. 2:**
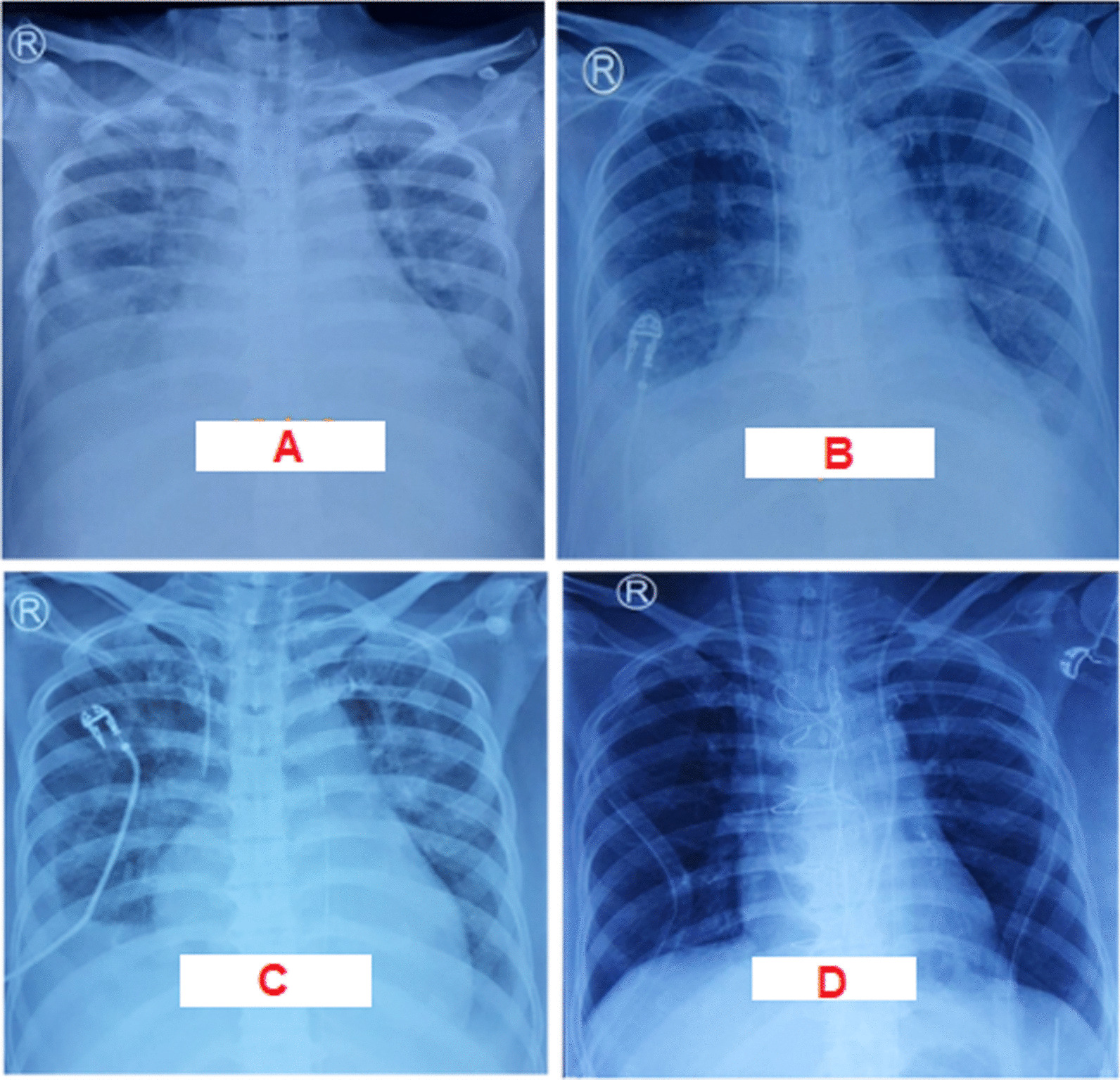
Chest radiograph evaluation on: **A** admition (first day) it showed congestive lungs with bilateral pleural effusion; **B** a week of hospitalization (seventh day) the sign of lungs congestion was still remain despite adequate diuresis; **C** second day of IABP implantation (eleventh day) the congestion remarkably reduced and heart border became more apparent; **D** second post-operative day (14th day). The yellow arrow indicates the tip of IABP

The ECG was sinus rhythm with poor R wave progression at precordial leads as well as biphasic and inverted T waves at leads V1 to V3 that may suggest a late onset anteroseptal myocardial infarction (Fig. 1). There was no clear information regarding the exact onset of acute coronary syndrome. Chest radiography displayed pulmonary congestion and pleural effusion of both lungs (Fig. 2A). Initial laboratory examination showed mild anaemia (haemoglobin of 10.9 g/dL and haematocrit of 31%) and hypoalbuminemia (2.46 mg/dL). High-sensitivity cardiac troponin I was normal. Other laboratory indices included liver function tests, renal function tests, arterial blood gas parameters, and electrolyte levels were unremarkable.

**Fig. 3:**
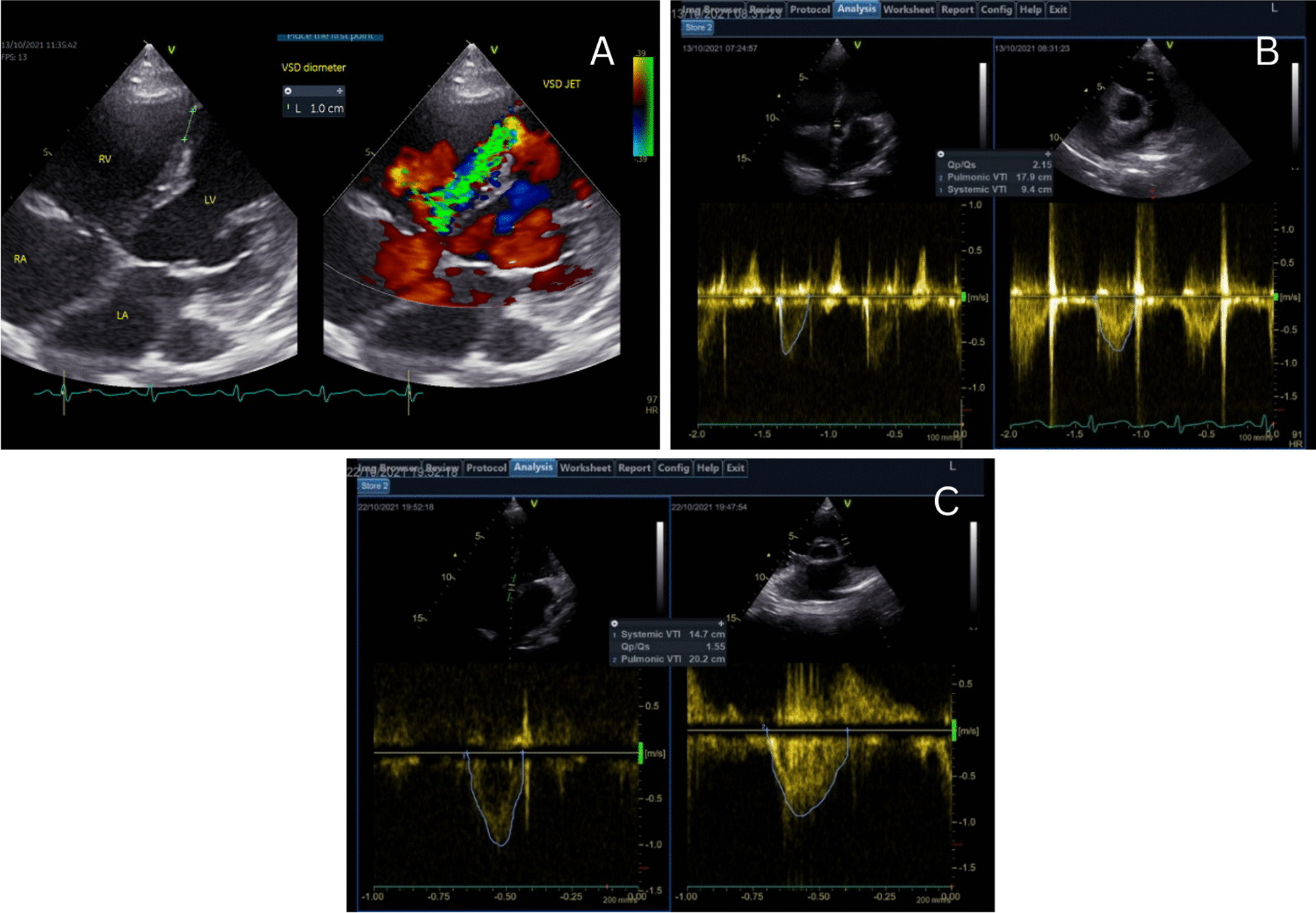
**A** ventricular septal defect with a diameter of 10 mm; **B** Qp/Qs ratio on the first day on dobutamine was 2.1 **C** on the second day of IABP without dobutamine, the Qp/Qs ratio was 1.55. This indicated an effective shunt reduction using IABP

Echocardiography revealed hypokinetic anteroseptal and anterior segments of the left ventricular (LV) wall, while other segments’ motion remained normal. The LV ejection fraction (LVEF) was 50%. A left-to-right shunt was detected from a ventricular septal defect of 10 mm in size at the apical portion of the interventricular septum (Fig. 3A). The non-invasive hemodynamic assessment showed reduced cardiac output (CO), cardiac index (CI), LV outflow tract velocity time integral (LVOT VTI), respectively 2.87 L/min, 1.81 L/min m^2^ and 10 cm (the normal values are respectively 4–8 L/min, 2.8–4.2 L/min m^2^, and 20 ± 3 cm). The systemic vascular resistance (SVR) was relatively high (1997.7 dynes s/cm^5^). There was an increase in estimated right atrial pressure (RAP) (> 15 mmHg), LV filling pressure and pulmonary capillary wedge pressure (PCWP) (*E*/*e*’ ratio of > 14). The pulmonic to systemic blood flow (Qp/Qs) ratio was 2.1 (Fig. 3B). Lung ultrasonography (LUS) displayed pulmonary congestion and bilateral pleural effusion in the right and left costophrenic sinuses.

The patient was transferred to the intensive cardiac care unit (ICCU). The objectives were to improve the clinical status and preparation for coronary angiography. Dobutamine (5 mcg/kg/min) was administered to maintain adequate perfusion. Norepinephrine was safely tapped off, followed by the introduction of a low-dose ACE inhibitor, Ramipril 2.5 mg. Furosemide was administered through continuous infusion to achieve adequate decongestion. The dose was started by 10 mg/hour and gradually lowered following the improvement in patient’s volume status. Diabetes mellitus was treated with insulin, and comorbidities were treated accordingly.

During the first week in the ICCU, the urine output was adequate with diuretic and inotrope support (the total urine output was 17.7 L and the total water deficit was 11.3 L). However, the heart failure symptoms remained, and the echocardiography hemodynamic monitoring showed only slight improvement in CO, CI, and LVOT VTI. The LV filling pressure and PCWP were still high with an *E*/*e*’ ratio of > 14. Since inotrope can enhance left-to-right shunting through the septal defect, lowering the dose may reduce the shunt. However, several attempts to lower the inotrope dose resulted in hypotension, decreasing urine output, and worsening symptoms of congestion. The patient clinical profile may be described as “stable but inotrope dependent”. It took ten days for the patient to tolerate the supinated position without developing severe symptoms and to be considered eligible for a coronary angiography procedure. Within this duration, there were episodes of hypokalaemia and metabolic alkalosis secondary to loop diuretic therapy.

**Fig. 4:**
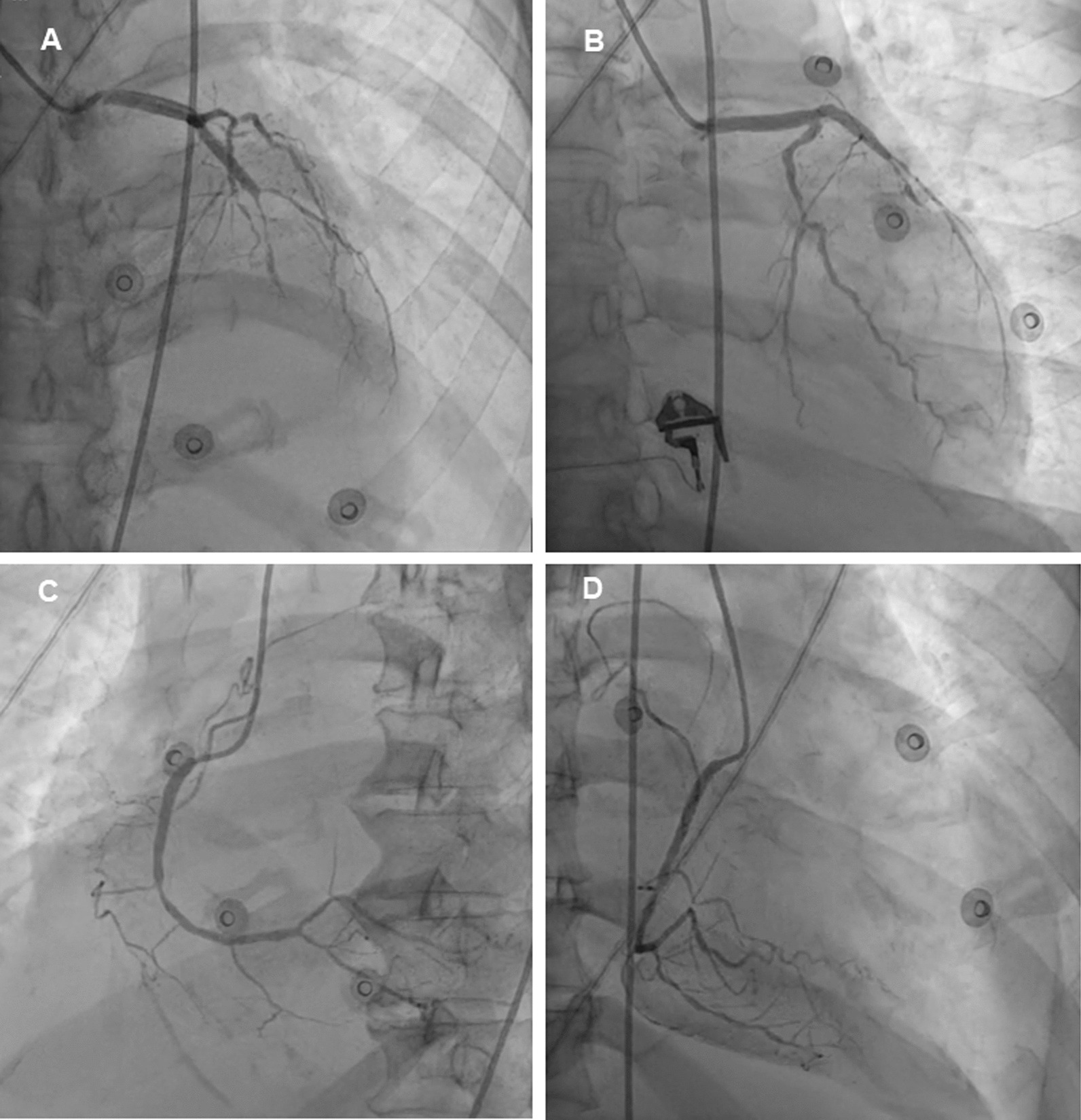
Invasive coronary angiography revealed: **A** and **B** a normal left main coronary artery, critical stenosis at mid left anterior descending artery, total occlusion at distal left artery descending, and 50% stenosis at mid left circumflex artery; **C** and **D** A significant 80% stenosis at mid right coronary artery

Coronary angiography was performed on the tenth day of hospitalization. It revealed a chronic total occlusion of the left anterior descendent artery, up to 50% stenoses at distal portion of left circumflex artery, and severe stenoses of the right coronary artery (Fig. 4). The Heart Team unanimously decided that surgery would be performed after preoperative optimization. IABP was implanted as a bridge to surgical therapy. There were significant improvements in symptoms as well as hemodynamic parameters following the application of IABP (1:1 augmentation ratio). On the second day of IABP utilization, the inotrope was completely tapped off. Hemodynamic monitoring showed left-to-right shunting reduction (Qp/Qs ratio 1.55) (Fig. 3C) and significant augmentation in CO, CI, and LVOT VTI (respectively 4.14 L/min, 2.67 L/min m^2^, and 14.9 cm). There was also improvement in ventricular filling pressure and PCWP (*E*/*e*’ ratio < 14).

**Fig. 5:**
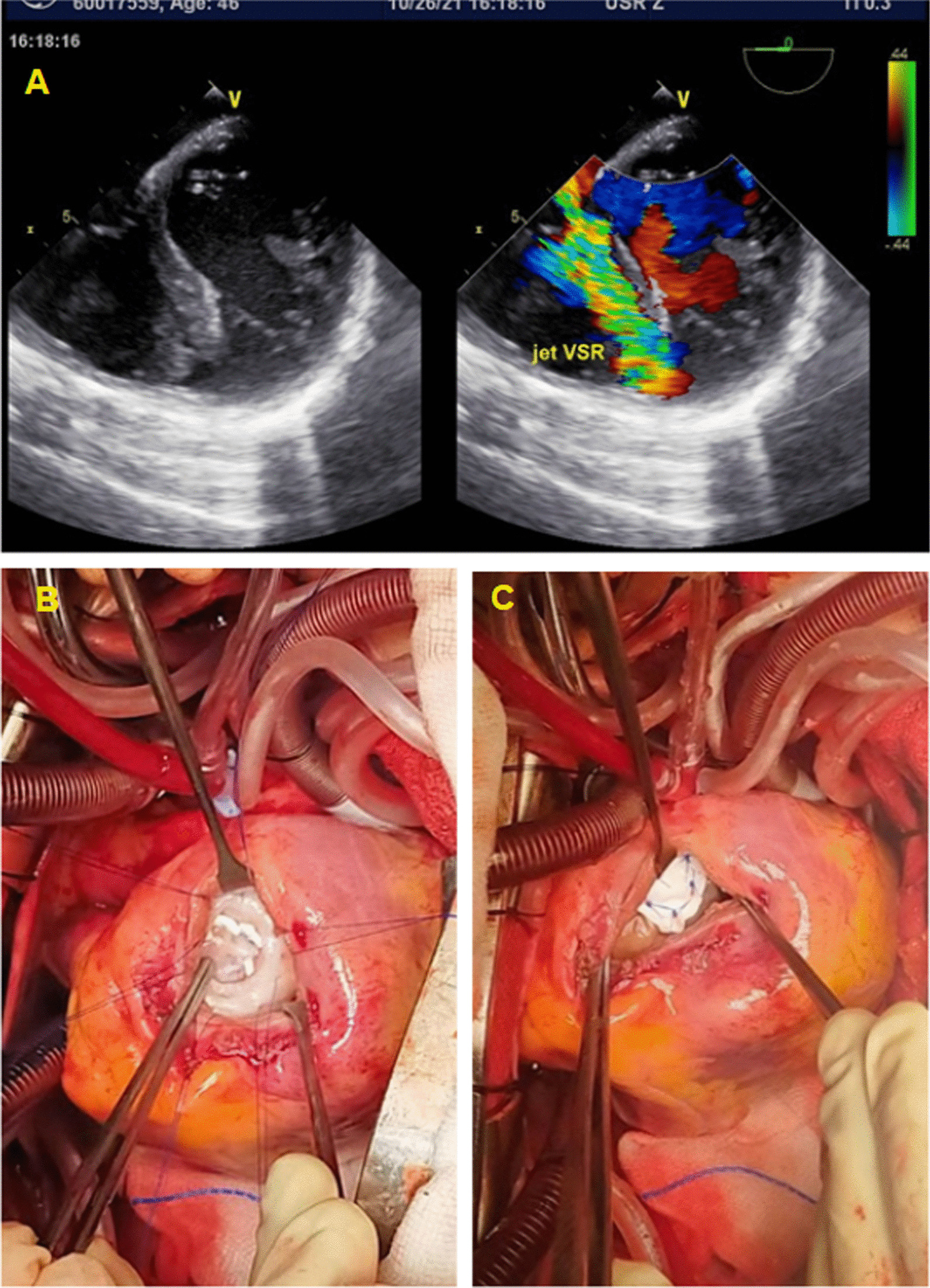
**A** Trans-oesophageal echocardiography showed a ventricular septal defect with diameter of 15 mm; **B** Left ventriculotomy revealed a ventricular septal defect of 15 mm in diameter; **C** the ventricular septal defect was closed with polytetrafluoroethylene patch

The surgery was performed four days after IABP implantation. Pre-procedural trans-esophageal echocardiography (TEE) showed a defect at the apical portion of the ventricular septum with a diameter of 10 mm (Fig. 5A). The surgery procedures included coronary artery bypass grafting (CABG) and VSR closure. During CABG, anastomoses were made from the Saphenous vein graft (SVG) to the right posterior descending artery (RPDA) and from Left Internal Mammary Artery (LIMA) to the middle segment of the Left Artery Descending (LAD) artery. A septal defect of 15 mm in diameter was revealed during left ventriculotomy (Fig. 5B). It was then closed with a polytetrafluoroethylene patch*.* Post-procedural TEE showed no residual flow through the septal patch.

Postoperative care in the intensive care unit was uneventful. The IABP was still maintained after the surgery and safely removed after proper weaning with minimal vasoactive drugs on the third postoperative day. The patient was transferred to the low care unit on the fifth postoperative day and was discharged after several days without relapsing symptoms of heart failure.

## Discussion

VSR is a rare but devastating complication of AMI. Its prevalence has substantially declined in the recent era of early reperfusion therapy, ranging between 0.17 and 0.31% [9–11]. It is associated with extensive comorbidities, resulting in poor cardiac output, multiorgan failure and death. Despite improvement in early diagnosis and treatment, the current mortality rate remains high (41–80%) [9] and it is extremely high (> 90%) among patients whose VSR is left untreated. [11]

Hemodynamically, the occurrence of VSR gives rise to left-to-right shunt and reduces forward blood flow. Compensatory increase in systemic vascular resistance, thus the LV afterload, leads to an even more rigorous left-to-right shunt [18]. The management should be directed at reducing the vicious left-to-right shunt with afterload-reducing pharmacological agents and MCSs [9, 14]. Inotropes and vasopressors may worsen left-to-right shunting, whereas vasodilators decrease shunting at the expense of hypotension [19]. Our patient, in this case, was so prone to hypotension that a vasodilator agent such as nitroprusside or nitroglycerin was not a safe option.

Several studies have demonstrated IABP's beneficial hemodynamic effects in post-MI VSR [19–22]. Its application results in a significant reduction in left-to-right shunt volume and shunt flow ratio, with a concomitant increase in effective systemic CO [20]. These outcomes are mainly achieved from afterload reduction [14, 21]. IABP also increases coronary perfusion and decreases myocardial oxygen consumption. Both effects result in the amelioration of myocardial ischemia [23–26].

The current guidelines suggest urgent surgical repair as the definitive treatment for post-MI VSR, but there is no consensus on the optimal timing of the surgery [8]. In all patients with severe heart failure that do not respond rapidly to aggressive heart failure therapy, early surgery should be performed [8]. However, delayed surgical repair may be considered in those who respond well to aggressive therapy. Delayed surgery is beneficial as it allows infarcted tissue maturation and firm scar formation for better anchoring of suture and patch material [12]. Nonetheless, this strategy also carries the risk of rupture extension and death while waiting for surgery [8]. Preoperative optimization of the already poor cardiac function and hemodynamic status during the waiting periods is challenging [27]. MCSs utilization, either as a sole device or in combination, is often required. [15]

Kettner et al. discovered that IABP support in shock caused by post-MI mechanical complications—primarily due to VSR—significantly reduces preoperative mortality [22]. Furui et al. reported that surgery could be delayed for an average of 9 days from acute VSR onset using IABP or respiratory management without deterioration of organ function [28]. The strategy was also associated with favorable 30-day mortality and long-term outcome. However, IABP hemodynamic support is often insufficient in more critical patients, and combination with other MCS may be beneficial [29]. Morimura et al. reported that a combination of IABP and venoarterial extracorporeal membrane oxygenation (VA ECMO) during preoperative optimization might improve outcomes of patients with post-MI VSR complicated by cardiogenic shock [30]. 33 It is worth mentioning that transcutaneous VSR closure has been proposed as a preoperative strategy prior to definitive surgical repair to reduce shunt and thereby stabilize the patient’s clinical condition [15, 31]. However, it adds up the potential risk of complications such as device dislodgement and hemolysis [32, 33].

Our patient presented with late AMI and was revealed to have a severe two-vessel coronary artery disease that required CABG revascularization. A meta-analysis that included 9212 patients undergoing CABG showed that preoperative implanted IABP was associated with a mortality relative risk reduction of more than 4% [34]. There were also reductions in the risk of MI and renal failure. Additionally, intensive care and total hospital stays were significantly reduced, indicating possible health and economic benefits [34]. Yang et al*.* reported a study of 416 patients with LV dysfunction undergoing off-pump CABG that showed preoperative IABP was linked to lower 30-day mortality. [35]

## Conclusion

Despite the declining trend of IABP use in cardiogenic shock complicating AMI, IABP is still the first-line MCS in the case of post-MI VSR. Its use during the preoperative optimization period is associated with survival benefits in patients who were planned for delayed surgical repair of VSR and CABG. However, its hemodynamic support is insufficient in more critical patients. This situation may call for more advanced measures such as IABP combination with the other type of MCS or preoperative transcutaneous closure of VSR.

## Data Availability

Not applicable.

## Abbreviations

IABP: Intra-aortic balloon pump
MCS: Mechanical circulatory support
ACS: Acute coronary syndrome
AMI: Acute myocardial infarction
RCT: Randomized clinical trial
VSR: Ventricular septal rupture
CABG: Coronary artery bypass grafting
HR: Heart rate
BP: Blood pressure
RR: Respiratory rate
CO: Cardiac output
CI: Cardiac index
LVOT VTI: Left ventricle outflow tract velocity time integral
SVR: Systemic vascular resistance
RAP: Right atrial pressure
PCWP: Pulmonary capillary wedge pressure
Qp/Qs: Pulmonic to systemic blood flow
